# Efficacy and safety of intraocular lens removal combined with vitrectomy for acute endophthalmitis in pseudophakic eyes

**DOI:** 10.1007/s00417-026-07185-5

**Published:** 2026-03-21

**Authors:** Shady Suffo, Muhammad Bazido, Berthold Seitz, Alaa Din Abdin

**Affiliations:** Department of Ophthalmology, Saarland University Medical Center UKS, HomburgSaar, Germany

**Keywords:** Endophthalmitis, Intraocular lens removal, Pars plana vitrectomy, Surgical complications

## Abstract

**Purpose:**

To evaluate the efficacy and safety of intraocular lens (IOL) removal during vitrectomy in the treatment of primary endophthalmitis in pseudophakic eyes.

**Methods:**

A comparative retrospective study was conducted on 55 eyes diagnosed with endophthalmitis at the Department of Ophthalmology, Saarland University Medical Center, between the years 2016 and 2020. The study assessed both functional and morphological long-term outcomes in patients undergoing pars plana vitrectomy (PPV) with IOL removal versus those with IOL preserved. Key parameters measured included best corrected visual acuity, duration of hospitalization, and the rate of inpatient and post-discharge complications.

**Results:**

The removal of the IOL did not significantly affect treatment outcomes, as there were no notable differences in best corrected visual acuity, hospitalization duration and the postoperative complication rates between both groups.

**Conclusion:**

IOL removal does not seem to offer additional advantages in the management of endophthalmitis. Thus, PPV IOL preservation seems to be a safe approach. Retaining the IOL may represent a viable and safe option, avoiding unnecessary surgical intervention in acute settings without significantly compromising patient outcomes. However, larger studies are required to confirm these findings.

## Abstract (highlights)

**Key messages:**

*****What is known***:**

- Endophthalmitis is a devastating condition with outcomes that vary depending on the etiology and many prognostic factors.
- An intraocular lens is confirmed to be a point of adhesion and replication of causing organisms in endophthalmitis.

*****What is new***:**

- This study corroborates previous reports indicating that, in selected cases of endophthalmitis managed with timely vitrectomy and intravitreal antibiotics, preservation of the intraocular lens does not worsen final visual acuity.
- These data further refine existing evidence by showing that long‑term complication rates are comparable whether the IOL is preserved or removed, supporting a more individualized approach to IOL removal based on intraoperative considerations rather than presumed prognostic benefit.

## Introduction

Endophthalmitis refers to intraocular bacterial or fungal infection, involving the vitreous humor. Most cases are exogenous, with organisms introduced via trauma, surgery, or an infected cornea. Conversely, endogenous endophthalmitis occurs when the eye is seeded via the bloodstream [1–3].

Symptoms include red eye, pain, blurred vision, and swollen lids. Signs include reduced red reflex, cells in the anterior chamber, hypopyon, corneal edema, redness, eyelid swelling, conjunctival chemosis, fibrin in the anterior chamber, progressive vitritis, and increased intraocular pressure. Ocular ultrasonography is routinely performed to assess the diagnosis in case of media opacity [4–6].

Endophthalmitis is typically treated with pars plana vitrectomy (PPV) and intravitreal injection of antibiotics. The Early Vitrectomy Study (EVS) outlined the treatment of endophthalmitis worldwide, but the choices differ widely between clinicians and medical establishments due to the aggressive nature of the infection and the clinical uncertainties that remain unresolved in medical literature [4, 7, 8].

Numerous published studies have demonstrated that intraocular lenses (IOLs) can act as potential sites for bacterial adhesion, both in vitro and in vivo [9–13]. These studies have shown that cultures of removed IOLs from patients with endophthalmitis frequently yield positive results for causative organisms, indicating that the IOLs themselves may harbor organisms contributing to persistent or recurrent infections [9, 10, 12]. A number of these patients experience recurrent episodes of inflammation, which can persist for several months. These inflammatory episodes often continue until the IOL is surgically removed, suggesting a direct link between the presence of the IOL and the persistence of infection [14]. For example, Abdin et al. published a case report of recurrent post-cataract fungal endophthalmitis after intravitreal injections of Bevacizumab, where the patient exhibited long-term stability and absence of inflammatory episodes only following the removal of the IOL [15]. This observation supports the hypothesis that IOL removal can be a beneficial intervention in managing endophthalmitis [16]. However, it is important to note that recent studies have raised questions about the necessity and efficacy of IOL removal in treating this condition [17]. Hui et al. suggest that the removal of the IOL may not play a significant role in the resolution of the infection or the improvement of clinical outcomes [18].

In this study, we monitored endophthalmitis patients who were admitted to the Department of Ophthalmology at Saarland University Medical Center, evaluated their clinical findings, and assessed the results of therapy. We also compared the treatment outcomes for the pseudophakic patients who underwent IOL removal during their first treatment with those who did not.

## Patients and methods

This retrospective single-center study encompassed 55 eyes of 55 patients diagnosed with endophthalmitis and admitted to the Department of Ophthalmology at Saarland University Medical Center. The patients were treated during hospitalization and subsequently followed for 12 months’ post-discharge.

Endophthalmitis diagnosis was based on clinical symptoms (redness, pain, reduced vision), slit-lamp examination (marked anterior chamber reaction, vitreous haze or opacities, hypopyon), and ultrasound in case of media opacity), and both culture-positive and culture-negative cases meeting these criteria were included. Best corrected visual acuity (BCVA) was measured using Snellen charts and converted to the logarithm of the minimum angle of resolution (logMAR). Macular edema (ME) was evaluated using optical coherence tomography (OCT) (Spectralis, Heidelberg Engineering, Germany).

In this study, all patients presenting with intraocular inflammation that significantly obscures fundus visualization or with acute, severe vision reduction underwent PPV within 6 h of admission. The surgical procedure included an intraoperative core and posterior vitrectomy, complete removal of vitreal infiltrates, and anterior chamber washing to eliminate hypopyon and fibrin as well as reducing the pathogen load. All patients admitted in 2016 and 2017 underwent IOL removal as part of their primary surgical treatment. However, starting in 2018, there was a strategy change in the clinic, and primary IOL removal was no longer performed in cases of endophthalmitis. Instead, only surgical posterior capsulotomy and irrigation of the capsular bag were carried out. Samples of vitreous and excised tissues were sent for microbiological testing. There was a change in the primary operating surgeon between the two study periods; however, all surgical platforms, vitrectomy machines, and visualization systems remained consistent. Three surgeons participated in the procedures: one senior consultant and two consultants. The individual identity or level of surgical experience was not analyzed as a variable. All surgeons used a 23-gauge pars plana vitrectomy technique both before and after 2017. The same vitrectomy platform (DORC EVA with TDC technology) was used throughout the entire study period.

Postoperatively, patients initially received intensive wide spectrum topical antibiotic therapy, for example Moxifloxacin or combined preparation (Polymyxin-B-sulfat, Neomycinsulfat and Gramicidin) and then adjusted analogous to antibiogram results once available. Intravitreal injections of vancomycin (1 mg/0.1 mL) and ceftazidime (2.2 mg/0.1 mL) were administered in a predefined regimen of three injections given every other day, specifically on postoperative days 2, 4, and 6. This schedule was applied uniformly throughout the entire hospitalization, and the maximum number of injections per treatment cycle was capped at three. Additionally, local and systemic steroids were routinely used. Intravenous antibiotic therapy was applied following the Magdeburg three-step plan for systemic antibiotic therapy according to Prof. Behrens-Baumann [19]. Anterior chamber washing was repeated as necessary in response to increased inflammation signs in the anterior chamber (cells and fibrin). Keratoplasty was performed in patients with infectious keratitis as a reason for endophthalmitis.

Patients were discharged once the intraocular inflammation was significantly reduced, defined by the absence of vitritis and hypopyon. Enucleation was performed in cases resistant to therapy or recurrent cases in blind, painful eyes. Globe loss, defined as enucleation or evisceration of the study eye at any time during follow‑up, was explicitly classified as treatment failure.

Inclusion Criteria:

1. Eyes diagnosed with acute endophthalmitis
2. Pseudophakia
3. No prior primary treatment for endophthalmitis

Exclusion Criteria:

1. History of endophthalmitis in the subject eye
2. Inability or refusal to follow-up through the acute phase of treatment

Primary Outcome Measures Included:

1. BCVA, measured via Snellen charts, and converted to logMAR
2. Duration of hospitalization
3. Number and type of surgical interventions during hospitalization and within 12 months post-discharge
4. Complications during hospitalization and within 12 months post-discharge like retinal detachment, epiretinal membrane, vitreous hemorrhage, and recurrence of endophthalmitis

The data was recorded at admission, discharge, and at 6 weeks, 6 months, and 12 months post-discharge. Additionally, when available, the last recorded values prior to the inflammatory event (pre-admission) were utilized.

## Statistical analysis

Statistical analysis was performed using IBM SPSS Statistics V.28. Results were considered statistically significant if the *p* value was < 0.05.

This study employed a multifaceted statistical approach to analyze the outcomes of multiple ophthalmic interventions among 55 participants. Descriptive statistics present demographic and clinical characteristics. The analysis of BCVA (logMAR) measurements over six follow-ups utilized ANOVA to evaluate the changes over consecutive follow ups in BCVA and to compare BCVA changes between the participants with IOL removal and IOL preservation. Normality of continuous outcomes (BCVA) was assessed using Shapiro–Wilk, Anderson–Darling, skewness and kurtosis tests; all four indicated non‑normal distributions (*p* < 0.05 for all variables). Therefore, in addition to classical univariate ANOVA, a nonparametric aligned rank transform (ART) ANOVA with ART‑based Tukey post hoc tests was performed for BCVA.

Pearson’s chi-square test evaluated associations between IOL removal and the number of ophthalmic interventions during hospitalization.

Because prognosis in endophthalmitis depends strongly on the causative organism, visual acuity over time (logMAR) was additionally analyzed in subgroup models stratified by pathogen using ART-ANOVA (Gram positive vs Gram negative, bacteria vs. fungi)

## Results

During the study period, 112 patients were admitted to the Department of Ophthalmology at Saarland University Medical Center with a diagnosis of endophthalmitis. Of these, 55 patients met the inclusion criteria for the study. Patients were divided into two groups based on their initial treatment:

1. Patients who underwent intraocular lens (IOL) removal as part of their initial treatment (*n* = 23, 41.8%)
2. Patients who did not undergo IOL removal (*n* = 32, 58.2%)

Statistical analysis (Table 1) revealed an age distribution ranging from 13 to 92 years, with an average age of 70.5 years. Gender distribution among the participants was as follows: 21 men (38.2%) and 34 women (61.8%). Regarding laterality, the right eye was affected in 26 participants (47.3%), and the left eye was affected in 29 participants (52.7%). In terms of comorbidities, diabetes mellitus was present in 23.6% of the participants, arterial hypertension in 47.3%, and immunosuppression in 1.8%.

**Table 1:** Baseline characteristics and microbiological findings in 55 endophthalmitis cases, stratified by intraocular lens (IOL) removal status

Variable		All *n* = 55	With IOL-removal 23 (41.8%)	Without IOL-removal 32 (58.2%)
Gender	Male	21 (38%)	7 (30%)	14 (44%)
	Female	34 (62%)	16 (70%)	18 (56%)
Laterality	Right eye	26 (47%)	10 (43%)	16 (50%)
	Left eye	29 (53%)	13 (57%)	16 (50%)
Age (years)		70.5±15	69.4±13	71.3±15
Comorbidity	Diabetes mellitus	13 (24%)	7 (30%)	6 (19%)
	Immune suppression	1 (1.8%)	1 (4%)	0 (0%)
	Arterial hypertension	26 (47%)	13 (57%)	13 (41%)
Primary cause	Post cataract surgery	16 (29%)	8 (35%)	8 (25%)
	Post keratitis	24 (45%)	9 (39%)	15 (47%)
	Post intravitreal injection	8 (15%)	4 (17%)	4 (13%)
	Endogenous	2 (4%)	0 (0%)	2 (6%)
	post trauma	2 (4%)	1 (4%)	1 (3%)
	Post keratoplasty	3 (5%)	1 (4%)	2 (6%)
Microbiological etiology	Gram-positive cocci	28 (51%)	13 (56%)	15 (47%)
	Gram-negative bacilli	4 (7%)	1 (4%)	3 (9%)
	fungi	5 (9%)	1 (4%)	4 (13%)
	Gram-positive cocci and fungi	4 (7%)	1 (4%)	3 (9%)
	Gram-negative bacilli and fungi	1 (2%)	0 (0%)	1 (3%)
	No growth	23 (42%)	9 (39%)	14 (44%)

In this study, endophthalmitis due to various reasons was studied: postoperative procedures (16 cases), post-keratitis infections (24 cases), after intravitreal injections (8 cases), endogenous factors (2 cases), post-traumatic incidents (2 cases), and post-keratoplasty complications (3 cases).

Microbiological analyses were performed on biopsy samples. The results revealed that 28 specimens were positive for Gram-positive cocci, four for Gram-negative bacilli, and five for fungi. Additionally, four specimens demonstrated a co-infection with Gram-positive cocci and fungi, while one specimen exhibited both Gram-negative bacilli and fungi. No microbial growth or evidence of microorganisms was observed in 23 specimens.

An intervention analysis revealed that 23 patients (41.8%) underwent IOL removal, in contrast to 32 patients (58.2%) who received PPV with IOL preservation.

The research methodology included the analysis of 241 measurements across six distinct follow-ups to assess the impact on BCVA, uncovering significant changes of BCVA over the different follow-ups (*p* < 0.001) (Table 2).

**Table 2:** Changes of best corrected visual acuity (BCVA) measured with Logarithm of the Minimum Angle of Resolution (logMAR) over study follow-ups

BCVA	Total	IOL.Ex	No IOL.Ex	Mean difference (removal-preservation)	*P* value	Significant?
Before *N* = 33	1.18±0.14	1.32±0.20	1.03±0.18	–0.005	0.943	no
Admission *N* = 48	2.17±0.12	2.21±0.17	2.13±0.15	+0.010	0.742	no
Discharge *N* = 48	1.69±0.12	1.66±0.17	1.72±0.15	–0.006	0.814	no
6 Weeks *N* = 44	1.37±0.12	1.43±0.18	1.32±0.16	–0.002	0.958	no
6 Months *N* = 41	1.26±0.12	1.32±0.18	1.20±0.16	+0.012	0.866	no
1 Year *N* = 36	1.35±0.13	1.32±0.20	1.37±0.17	+0.021	0.784	no

*Before* Before admission, *Admission* at admission, *Discharge* upon discharge, *6 weeks *6 weeks post-discharge, *6 months* 6 months post-discharge, *1 year *1 year post-discharge, *IOL* intraocular lens, *IOL Ex *cases treated with intraocular lens removal, *no IOL Ex* cases treated without IOL-removal

Mean LogMAR scores were lowest prior to admission (*M* = 1.178), peaked at admission (*M* = 2.172), and then improved again at later follow-ups (upon discharge *M* = 1.694; 6 weeks post discharge *M* = 1.377; 6 months post discharge *M* = 1.263; 1 year post discharge *M* = 1.350).

We explain low pre-endophthalmitis BCVA in both groups as resulting from underlying conditions leading to endophthalmitis or necessitating interventions that predispose to infection, such as macular degeneration (common in repeated anti-VEGF injection cases), cataracts, corneal ulcers, and post-corneal transplantation states. These pathologies inherently impair baseline vision across cohort before hospital admission. Records show pre-endophthalmitis BCVA for 33 total cases.

**Fig. 1:**
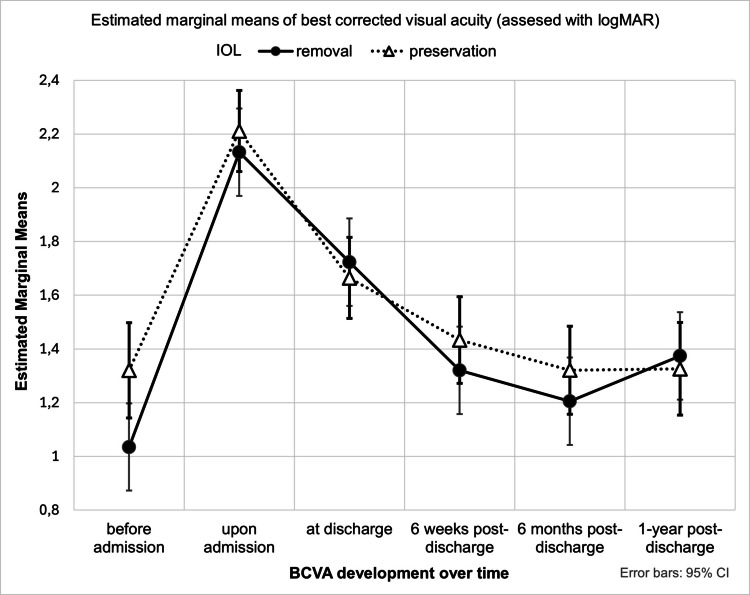
Changes of best corrected visual acuity (BCVA) over time in both study groups with intraocular lens (IOL) removal or preservation

These values illustrate the changes in BCVA, with comparisons involving the whole sample indicating significant differences in BCVA at various follow-ups of the study (Fig. 1)

The main effect of IOL removal was not significant (*p* = .422), with mean LogMAR scores of 1.465 (IOL preservation) and 1.546 (IOL removal). There was no significant difference in BCVA between the two groups at any of the six follow-ups (all *p* > 0.7). The two-way ANOVA confirmed a significant main effect of time (*p* < 0.001), but neither the main effect of IOL removal (*p* = 0.817) nor the interaction between time and IOL removal (*p* = 0.999) on BCVA reached statistical significance.

In the ART ANOVA, the time effect on LogMAR remained significant (*F*(5) = 11.02, *p* = 0.0001), whereas the main effect of IOL removal (*F*(1) = 0.84, *p* = 0.35) and the time × IOL removal interaction (*F*(5) = 0.22,* p* = 0.95) were not, confirming that the key time effects were robust to nonparametric re‑analysis.

In a sensitivity analysis restricted to the 23 culture-negative cases, time remained significantly associated with BCVA (*F* = 3.51, *p* = 0.005), whereas the main effect of IOL status (*F* = 0.99, *p* = 0.323) and the time × IOL removal interaction (*F* = 0.99, *p* = 0.429) were not statistically significant, indicating a similar pattern to the full cohort.

Pairwise comparison showed no significant difference in overall BCVA (*p* = 0.422), and duration of hospitalization between the two groups (Table 3).

**Table 3:** Comparison of parameters between both groups

Variables	Difference	*P* value
BCVA (logMAR)	0.81±0.10	0.422
Duration of hospitalization (days)	0.87±1.61	0.590

Best corrected visual acuity (BCVA) at all time points measured with logarithm of the minimum angle of resolution (logMAR)

In a sensitivity analysis excluding the single immunosuppressed patient (*n* = 54), the pattern and significance of the ART-ANOVA results for BCVA over time were unchanged, with a significant main effect of time (*F* = 10.939, *p* ≈ 0.002) and nonsignificant main and interaction effects of IOL removal (main effect *F* = 1.180, *p* = 0.278; time × IOL removal interaction *F* = 0.075, *p* = 0.995).

In subgroup analyses stratified by Gram stain, time remained significantly associated with BCVA (*F* = 11.72, *p* < 0.001), whereas neither the main effect of Gram category (*F* = 1.62, *p* = 0.20) nor the time × Gram interaction (*F* = 0.58, *p* = 0.82) was statistically significant. Likewise, when comparing infections as bacterial, fungal, or mixed, time showed a significant main effect on BCVA (*F* = 7.60, *p* < 0.001), while the main effect of pathogen group (*F* = 2.85, *p* = 0.06) and the time × bacteria interaction (*F* = 1.39, *p* = 0.19) were not significant.

Pearson chi-square test was utilized to examine the relationship between IOL removal and the number of ophthalmic interventions during hospitalization which included PPV, anterior chamber washing, intravitreal injections, keratoplasty, amniotic membrane transplantation, enucleation. It revealed no significant association between the number of interventions and IOL removal, with *p* values ranging from 0.22 to 0.81 (Table 4).

**Table 4:** Comparison of interventions during hospitalization between both groups

Intervention	Without IOL-removal *n* = 32		With IOL-removal *n *= 23		*P *value
	Absolute	mean	Absolute	mean	
Pars plana vitrectomy	32 (100%)	1.43±0.6	23 (100%)	1.34±0.6	0.26
Intravitreal injections	32 (100%)	2.59±2.1	23 (100%)	3.1±2.6	0.57
Anterior chamber washing	20 (62.5%)	1.43±1.6	20 (87%)	1.78±1.4	0.46
Keratoplasty	11 (34.4%)	0.34±0.5	6 (26.1%)	0.30±0.6	0.32
Amniotic membrane transplantation	2 (6.3%)	0.06±0.2	4 (17.4%)	0.17±0.4	0.22
Enucleation	1 (3.1%)	0.03±0.2	1 (4.3%)	0.04±0.2	1.00

*P* value *p* (χ²): difference in distribution of interventions between IOL‑removal groups. *Absolute* Absolute number per group,* mean* mean±standard devation

Overall, 42 patients (77%) completed a single cycle of three postoperative intravitreal injections, while 13 patients (23%) underwent a repeated treatment cycle (pars plana vitrectomy with intravitreal injection followed by three further injections at 2‑day intervals) due to recurrence.

The statistical analysis conducted to evaluate the correlation between IOL removal and post-discharge complications revealed no associations (Table 5). No significant correlation was observed regarding the post-discharge complications, including epiretinal membrane, vitreous hemorrhage, and ME, all showing *p* values exceeding the 0.05 threshold, indicating a lack of statistical significance. A chi-squared test was performed to examine the association between IOL removal and recurrence. The test showed no significant relationship between the two variables (*p* = 0.639). Recurrence occurred in seven cases overall, with three in the IOL preservation group and four in the IOL removal group. Statistical power was low (0.14), suggesting that the study may be underpowered to detect smaller differences.

**Table 5:** Comparison of post discharge complications between both groups

Post-discharge complications	without IOL-removal *n* = 32	with IOL-removal *n* = 23	*P* value
Enucleation	3 (9.4%)	0 (0%)	0.26
Retinal detachment	3 (9.4%)	5 (21.7%)	0.26
Macular edema	3 (9.4%)	2 (8.7%)	1.00
Epiretinal membrane	1 (3.1%)	0 (0%)	1.00
Vitreous hemorrhage	1 (3.1%)	1 (4.3%)	1.00
Recurrence	3 (9.4%)	4 (17.4%)	0.64

*IOL* Intraocular lens

The Mann–Whitney *U* test comparing IOL-removal groups showed low power (0.11), indicating that the study was underpowered to detect small between-group effects. Similarly, the chi-squared test for the association between IOL-removal and time had very weak power (0.05). In contrast, the Kruskal–Wallis test for differences across time points demonstrated strong power (1.00), supporting the robustness of the significant changes over time observed.

## Discussion

Many studies have demonstrated the possibility of in vitro bacterial adhesion to IOL. Other research indicates that the removed lenses during the treatment of endophthalmitis are already colonized. This theoretically suggests:

1. IOL may serve as a source of possible reinfection, as it is already the carrier of causative organisms [9, 10, 13, 20, 21].
2. IOL can exacerbate ongoing inflammation, functioning as a replication site for bacteria and a source of persistent inflammation
3. A study from Schroeder et al. at the University of Saarland Hospital shows that IOL surface modifications modify Hydrophilicity considerably, and that the water content of acrylate materials does not predict the extent of surface hydrophilicity of acrylate materials. These results provide standardized information on IOL characteristics considered to be important for biocompatibility assessment [22].

It has also been suggested that routine doses of antibiotics cannot achieve the minimum inhibitory concentration in the IOL and capsule, leading to poor prognosis and frequent recurrence [23–25].

The removal of the IOL remains a controversial issue, with many publications discussing its necessity, especially if the capsular bag is affected [12, 15, 26]. Many studies show that PPV while preserving IOL is a safe and effective method for treatment, and that IOL removal is not a necessary component [17, 23]. However, endophthalmitis is often cited as one of the leading indications for IOL removal, especially for low-grade chronic endophthalmitis [27].

On the other hand, patients undergoing IOL removal will also need a subsequent procedure for secondary lens implantation to correct the refractive error.

Final visual acuity was an important measure in the Early Vitrectomy Study (EVS), where cases with initial light perception visual acuity achieved much better results after vitrectomy than the control group. Another study by Conrady et al. found that visual improvement plateaued in 67.7% of patients by 1 month after diagnosis [4, 8, 17].

The results of our analysis revealed a statistically significant increase in BCVA across the entire sample from admission onward, but there was no significant difference between the two groups in the overall BCVA values during the study period. This indicates that the removal of IOL did not affect the visual outcome (either positively or negatively). Furthermore, there was no statistically significant difference in BCVA between the first measurement (prior to infection) and BCVA 1 year after discharge. It should be noted, however, that baseline BCVA prior to infection was already very poor, which markedly limited the potential for further measurable deterioration. Thus, the lack of a statistically significant difference reflects that BCVA remained at a similarly low level over time rather than indicating a favorable visual outcome

Duration of hospitalization can be considered a predictive factor for the presentation of severe endophthalmitis, especially in middle-aged and elderly patients. A study by Regan et al. found that the average inpatient stay was 16.1±11.9 days [28, 29].

Our study did not find a significant difference in the duration of hospitalization or the number of inpatient ophthalmic interventions between the two groups.

Long-term complications after endophthalmitis are well documented and studied. A study by Lugo et al. classified visual acuity of no light perception, panophthalmitis, or exogenous etiology as risk factors for enucleation or evisceration. Another study by Wang et al. reported that, the incidence of retinal detachment after endophthalmitis was 14.8% (*N* = 16/108) and the presence of aphakia and posterior synechiae was associated with the development of retinal detachment in these cases [30, 31].

In our study, the rate of post-discharge complications did not significantly differ between the two groups. In addition, there was no significant difference in the development of macular edema or central macula thickness between both groups.

A study from shields et al. found recurrence in 12 patients in 535 studied cases. Another study from the Saarland University Medical Center described successfully treating a patient of recurrent endophthalmitis (Saccharomyces cervisiae fungi) with emergency anterior and posterior segment washout with IOL removal [15, 32].

The analysis did not demonstrate a significant difference in recurrence rates between patients who underwent IOL removal and those who did not. Although the recurrence appeared numerically higher in the IOL-Ex 1 group, this difference was not statistically significant. Importantly, the low power of the test indicates that the current sample size may be insufficient to reliably detect clinically meaningful differences. Larger studies with adequate sample sizes are therefore needed to clarify whether IOL removal has any impact on recurrence risk. These results indicate that the removal did not significantly contribute to the safety or efficacy of the treatment process. This aligns with the findings of other studies that demonstrate vitrectomy with IOL preservation is a safe treatment option, and that the IOL in most pseudophakic endophthalmitis cases can be safely preserved [17, 23].

The lack of significance may be related to limited statistical power, as confirmed by the post hoc analysis. Future studies with larger sample sizes are warranted.

Repeated intravitreal vancomycin was administered in this series without any vasculitic or occlusive complications compatible with vancomycin associated retinal vasculitis. This is consistent with pharmacokinetic data indicating that intravitreal antibiotics are cleared within a few days, with more rapid clearance in vitrectomized eyes, which provides a rationale for repeated injections to maintain therapeutic drug levels in non-resolving endophthalmitis. Experimental work in rabbit eyes has further shown that three intravitreal injections at 48-h intervals of a vancomycin–ceftazidime–dexamethasone combination at doses comparable to those used in humans are nontoxic, supporting the safety of repeated standard dose treatment [19, 33].

No alternative intravitreal antibiotics were employed; the vancomycin containing regimen reflects widely accepted first line therapy for acute postoperative endophthalmitis and aligns with established guideline-based practice patterns. At the same time, rare reports of vancomycin-associated hemorrhagic occlusive retinal vasculitis have raised awareness of a potential idiosyncratic risk, and patients in this series were therefore monitored for delayed onset retinal hemorrhages, vasculitis, or vascular occlusion, none of which were observed [19, 34, 35].

## Conclusion

In the treatment of pseudophakic patients with endophthalmitis, regardless of etiology, removal of IOL did not contribute to more favorable outcomes (increase in BCVA, shorter hospitalization), nor did it reduce the rate of post-discharge complications as presence of recurrent endophthalmitis. The results of the study suggest that IOL removal may be neither necessary nor beneficial in the treatment of first-time endophthalmitis.

## Main limitations

The study was limited by its retrospective nature, introducing bias from pre-existing data not initially intended for this research. Another limitation is the small sample size that limits the possibility for generalization of statistical results and may reduce statistical power, potentially overlooking significant effects.

Post hoc power analysis showed that the study had 11% power to detect the observed effect size between groups, suggesting that the study was underpowered for this outcome.

Although stringent clinical criteria were applied, culture-negative noninfectious cases cannot be completely excluded; however, the sensitivity analysis of culture-negative eyes showed a similar pattern of results, suggesting that this potential misclassification did not materially affect our conclusions.

## Data Availability

The datasets generated and analyzed during the current study are derived from patient medical records and contain sensitive clinical information. Therefore, they are not publicly available due to privacy and ethical restrictions. De-identified data may be available from the corresponding author upon reasonable request and with appropriate institutional approval.
